# HOW GROWING OLDER WITH THEIR “BEST FRIEND” CHANGES OLDER ADULT MEN’S PERSPECTIVES ON AGING

**DOI:** 10.1093/geroni/igae098.3275

**Published:** 2024-12-31

**Authors:** Ranell Mueller

**Affiliations:** Flathead Valley Community College, Kalispell, Montana, United States

## Abstract

Knowledge of the deeper meaning of how growing older alongside companion animal dogs affects men’s perspectives of aging is limited. This study employed qualitative research methods utilizing individual interviews and panel discussions with older adult men to investigate the dynamic phenomenon of their personal experience of growing older alongside their aging companion animal dogs. Individual audio-recorded and in-depth interviews and repeated panel discussions with a sub-group of participants, convened as a panel over a three-month period, explored behavioral and emotional manifestations of aging along with a companion animal. Analysis involved open, axial, and selective coding of transcripts to reveal underlying patterns within the data. Outcomes included insight into the role of dogs in the men’s perspectives of attitudes toward aging, toward not only themselves as older adult men, but also toward their aging dogs. Findings reveal that the older adult men felt empathy for their aging dogs, which translated to feeling empathy for themselves as well as other older adults. The men revealed attitudes of being sympathetic and understanding of the aging process simply because of aging alongside their aging dogs. A prominent idea that emerged was a feeling of grace toward oneself, others, and their aging dogs. This study offers insight into the deeper understanding of one effect caring for older companion animals has on older adult men’s attitudes toward aging. This study provides movement toward a theory of the role of dogs in the development of older adult men’s attitudes toward aging.

